# Two Decades of Arrayed Imaging Reflectometry for Sensitive, High-Throughput Biosensing

**DOI:** 10.3390/bios13090870

**Published:** 2023-09-05

**Authors:** Gabrielle Kosoy, Benjamin L. Miller

**Affiliations:** 1 Department of Biochemistry and Biophysics, University of Rochester, Rochester, NY 14526, USA; gabrielle_kosoy@urmc.rochester.edu; 2 Department of Dermatology, University of Rochester, Rochester, NY 14526, USA

**Keywords:** reflectometry, interferometry, protein microarray, antibody microarray, serology

## Abstract

Arrayed imaging reflectometry (AIR), first introduced in 2004, is a thin-film interference sensor technique that optimizes optical properties (angle of incidence, polarization, substrate refractive index, and thickness) to create a condition of total destructive interference at the surface of a silicon substrate. The advantages of AIR are its sensitivity, dynamic range, multiplex capability, and high-throughput compatibility. AIR has been used for the detection of antibodies against coronaviruses, influenza viruses, *Staphylococcus aureus*, and human autoantigens. It has also shown utility in detection of cytokines, with sensitivity comparable to bead-based and ELISA assays. Not limited to antibodies or antigens, mixed aptamer and protein arrays as well as glycan arrays have been employed in AIR for differentiating influenza strains. Mixed arrays using direct and competitive inhibition assays have enabled simultaneous measurement of cytokines and small molecules. Finally, AIR has also been used to measure affinity constants, kinetic and at equilibrium. In this review, we give an overview of AIR biosensing technologies and present the latest AIR advances.

## 1. Introduction

Over the past few decades, several label-free biosensing technologies have been developed based on operating principles of interferometry or reflectometry. These techniques, close relatives of spectroscopic ellipsometry, include arrayed imaging reflectometry (AIR) [1], biolayer interferometry (BLI) [2,3], the interferometric reflectance imaging sensor (IRIS) [4,5], reflectometric interference spectroscopy (RIfS) [6], 1λ reflectometry [7], oblique-incidence reflectivity difference microscopy (OI-RD) [8,9], and total reflectometric interference spectroscopy (TRIS) [10]. A broad overview of these methods may be found in a recent review by Fechner et al. [11]. 

**Figure 1:**
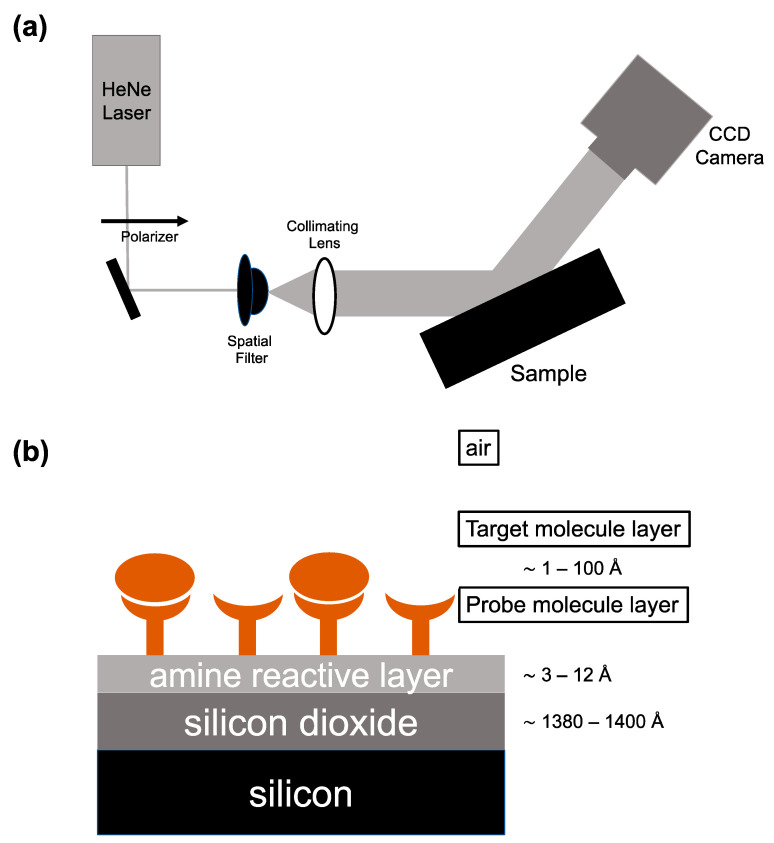
Arrayed imaging reflectometry (AIR). (**a**) Schematic of the essential elements of an arrayed imaging reflectometry apparatus; (**b**) AIR substrate. The thicknesses of the silicon dioxide and amine-reactive adhesion chemistry layers are chosen in order to produce a near-perfect antireflective condition upon attachment of probe molecules at defined spots on the array.

This article focuses on arrayed imaging reflectometry as developed in our laboratory, with a particular concentration on its recent applications in understanding the human response to infectious disease. AIR uses a HeNe laser (632.8 nm), linearly polarized to a high s:p ratio, as its light source. The polarized beam goes through a spatial filter, is expanded, and is then collimated before hitting the sensor chip at a 70.6° angle. This angle was determined computationally as providing the minimum reflective condition for the Si/SiO_2_ materials system and is precisely obtained experimentally either using standard optical rotation stages or a fixed-angle stage manufactured to the proper angle. A CCD camera is used to collect light reflected off the chip (Figure 1A). The sensor itself is a silicon/silicon dioxide substrate that is chemically functionalized with an array of capture probes chosen to bind biological molecules. Along with the choice of the 70.6° angle of incidence, by tuning the thickness of the surface oxide, adhesion chemistry, and spotted capture molecules, a condition of near-total destructive interference is created. Surface oxide and adhesion chemistry thicknesses only need to be optimized once for a particular sample matrix (i.e., human serum) since nonspecific binding to the background will be consistent with a first approximation. Individual spotted probe molecules can be optimized easily for thickness (achieving close to zero reflectivity) and uniformity by systematic variation of spotted probe concentration, buffer pH, and buffer additives in an array. As molecules bind to spotted probes, the optical thickness increases, degrading the antireflective condition and causing an increase in reflected intensity [1] (Figure 1B). The measured pixel intensity from light reflected off the surface of the sensor can be converted to a thickness in Ångstroms based on a model developed using reference substrates and spectroscopic ellipsometry. These thickness values are used to describe biosensing binding events at the surface of the AIR chip [12]. Variation in exposure time allows for thickness changes of 0.1 Å (average thickness change over the area of the sensor “seen” by 1 pixel of the CCD) to be resolved, with an upper limit of quantifiable thickness change of around 100 Ångstroms. 

While AIR is often used in a qualitative or semiquantitative mode, fully quantitative analysis is possible by correlating the observed thickness changes to concentration based on a calibration curve developed with samples of known concentration. A key advantage of AIR used in this fashion is that the instrument has no moving parts, does not require scanning, and (unlike plasmonic techniques) has no significant temperature sensitivity. In principle, AIR is also more sensitive than other interferometric techniques because it measures a change relative to a zero or near-zero condition rather than measuring a shift in a nonzero response. In practice, materials variation and sample noise (particularly nonspecific binding) may in some cases prevent achievement of optimum sensitivity. Of course, the use of a single incident wavelength places constraints on the materials system as discussed above; multiwavelength operation is also possible, however.

Since the first publication introducing the AIR technique in 2004 (then called “Reflective Interferometry”), the technology has been employed in a broad range of biodetection applications. As a label-free sensing platform, essentially any class of probe molecule may be arrayed on an AIR chip for detecting targets of interest. For example, arrays of antibodies against inflammatory biomarkers were shown to bind cytokines with lower limits of detection in the 1–100 pg/mL range [13]. AIR arrays employing biotin-conjugated aptamers as well as biotinylated antibodies demonstrated the potential of mixed probe surfaces [14]. Carbohydrates may also be used as probes in AIR arrays; for example, polymer-conjugated glycans were shown to be effective as surface probes to differentiate between human and animal influenza viruses, which have preferred binding to either NeuAcα2,6Gal or NeuAcα2,3Gal, respectively [15]. AIR was also used to detect IL-6 (via direct assay) and darunavir (via competitive inhibition) simultaneously [16]. 

In this article, we will highlight a few examples of AIR’s ability to address key bioanalytical problems. The technique has been used to measure antibody binding to a multitude of bacterial and viral antigens, and, conversely, arrays of human antibodies have been employed in rapid viral serotyping. These applications will be our primary focus. We will first discuss avian influenza virus antibody detection. The vast diversity of influenza viruses makes serological analysis challenging for traditional single- or low-plex techniques, particularly where sample availability is constrained, as in avian and animal surveillance. This is also true for other respiratory viruses, including SARS-CoV-2. The need to profile the human response to infection or vaccination against upper respiratory viruses, as well as to serotype the viruses themselves, seems a natural fit for AIR given its ability to achieve high multiplexing and low sample-volume requirements.

H5N1 strain influenza antigen arrays were used to detect antibody binding in human samples enrolled in an H5N1 vaccination trial [17]. A similar array provided information about the response of mallards in an influenza challenge experiment and enabled rapid analysis of field-acquired bird serum samples in the context of influenza surveillance [17]. Expanded arrays that were focused on upper respiratory pathogens (including SARS-CoV-2) proved highly useful in understanding the human immune response to the COVID-19 virus. Not limited to viral antigens, though, arrays of autoantigens were used to detect autoantibodies in human donor serum samples [18], and AIR arrays of *Staphylococcus aureus* (SA) bacterial antigens were able to discern SA-positive and SA-negative human samples [12]. Finally, we will discuss an aqueous version of AIR developed to provide multiplex kinetic measurements [19] and a new approach to measuring equilibrium (thermodynamic) affinities using an automated, high-throughput commercial version of AIR.

## 2. Sensing Immune Response to Influenza Antigens

The H5N1 avian influenza strain has already crossed over to humans, and there is interest in having systems in place in case of an outbreak. In a 2010 paper, an AIR array of influenza surface protein hemagglutinin (HA) was created to sense the immune response of humans immunized in H5N1 vaccination trials [20]. The AIR array incorporated recombinant hemagglutinin proteins (the primary vaccine antigen) from H1/New Caledonia/1999, H3/Wyoming/2003, H6/Teal/Hong Kong/1997, H5/Hong Kong/1997, H5/Hong Kong/2003, and H5/Vietnam/2004. The arrays were incubated with six clinical patient samples. The samples were provided by an H5N1 vaccine trial at the University of Rochester where patients were immunized with H5N1 or placebo. Serum samples showed binding to the H5 HA proteins as well as to H1. It was not surprising to find an H1 response since this paper was published soon after the 2009 H1N1 pandemic and the pandemic strain was included in the influenza vaccine formulation in 2010 [21]. The change in binding from pre- to postvaccination was shown to be similar to the difference in response between a placebo and nonplacebo patient sample. The United States continues to have programs that support preparedness for an avian flu pandemic and in particular continues surveillance and vaccine preparation for the H5N1 strain [22]. 

Building on this work, a 2015 paper demonstrated the utility of AIR in the context of influenza surveillance. An AIR array of hemagglutinin proteins from H1 to H12 and influenza B was used to examine mallard samples experimentally infected with influenza [17]. Several experimental improvements were implemented in this study relative to the 2010 work. First, all arrays were printed using piezoelectric spotting (Scienion S3) rather than via either a manual process or automated contact-based spotting. This provided a substantial improvement in chip-to-chip reproducibility, as well as overall spot morphology. Second, array responses were referenced to a nonreactive serum (FBS) negative control chip rather than to a buffer-only control. Mallards were challenged with H3N8, H6N2, and H12N8 influenza strains, and serum samples were collected 14 days post-challenge. Limits of detection (LOD) for each protein were determined by dilution curves with commercially purchased polyclonal antibodies. Thirteen mallard serum samples were tested on the arrays and a range of responses was observed, yielding data consistent with experimentally laborious singleplex assays. As a high-throughput method for assessing the response to a range of influenza antigens simultaneously, this array can serve as a surveillance tool for avian influenza. These are important data for helping understanding transmission [23]. 

While these applications demonstrated the ability of AIR antigen arrays to profile the immune response to influenza, arrays of anti-influenza human monoclonal antibodies can also be employed to serotype the virus. First introduced in 2018, this “Crowd on a Chip” approach easily discriminated among even closely related influenza strains using 85-plex to 115-plex arrays and was demonstrated to provide results analogous to whole-genome sequencing but in a much simpler, rapid format [24]. The first approach to discrimination used linear regression to describe the relationship between binding of human monoclonal antibodies (hmAbs) to two different influenza strains and identified outlier antibodies that were not as cross-reactive. Clustering techniques were also used to find which hmAbs cluster together when two vs. three clusters are set in the clustering algorithm. The approach also featured in a pandemic exercise conducted by the US National Institutes of Health, in which influenza researchers were charged with quickly developing a response to the possibility of canine influenza crossing over into humans. Here, the AIR “Crowd on a Chip” arrays were used to identify the vaccine strain most closely related to canine influenza and to suggest individual human monoclonals that might be studied further in the context of therapy [25].

## 3. Autoantibody Detection from Human Serum 

Autoimmune diseases such as Sjögren’s Disease and Systemic Lupus Erythematosus are diagnosed based on the pattern of antibody response to human antoantigens in a patient’s serum. In an effort to develop a rapid diagnostic test, an AIR array of five antigens of importance in both Sjögren’s Disease and Systemic Lupus Erythematosus was developed [18]. The antigens on the array, Ro60, La/SSB, Scl-70, Ro52, and BicD2, were formulated with different pH values and additives to optimize printing on the AIR chip. After achieving optimized print conditions (based on spot uniformity), activity of printed antigens was confirmed by exposing arrays to known polyclonal antibodies. The binding of autoantibodies to the array was then tested by incubating AIR arrays with known positive and other human donor serum samples at 0.1% and 5% dilutions. Overall qualitative responses from the AIR array were consistent with singleplex ELISA and multiplex Luminex assays. Subsequent serial dilutions on these samples showed LLOD and LLOQ values ranging from below 0.001% dilution to 0.5%, depending on the antigen.

## 4. Early Convalescent COVID-19 Sample Testing

With the onset of the COVID-19 pandemic in early 2020, previous work with AIR as a tool for assaying antiviral immune response suggested it could be rapidly adapted to examine SARS-CoV-2 response as well. Thus, a respiratory antigen AIR array to detect antibodies of relevance to COVID-19 was successfully developed within a few weeks, with the first manuscript published in 2020 [26]. This 14-plex array had antigens from HCoV-HKU, HCoV-229E, HCoV-OC43, MERS, SARS-1, SARS-2, influenza A, and influenza B. Polyclonal antibodies were used to successfully test antibody binding to SARS-2 antigens, providing initial validation of the array. Serum samples from 15 convalescent COVID-19 subjects were then tested. Experiments showed that at both a 1:5 and 1:20 dilution (performed for selected samples), antibody binding to SARS-CoV-2 antigens could be seen, and responses agreed with the results of singleplex ELISA assays for the same SARS-CoV-2 antigens. An experiment was conducted to show that AIR can be used to identify class-specific antibody binding for IgG and IgM with the use of a secondary antibody. The study found class-specific binding for both COVID-negative and -positive samples.

## 5. Commercialization of AIR and Its Use for Longitudinal Analysis of SARS-CoV-2 Immunity

**Figure 2:**
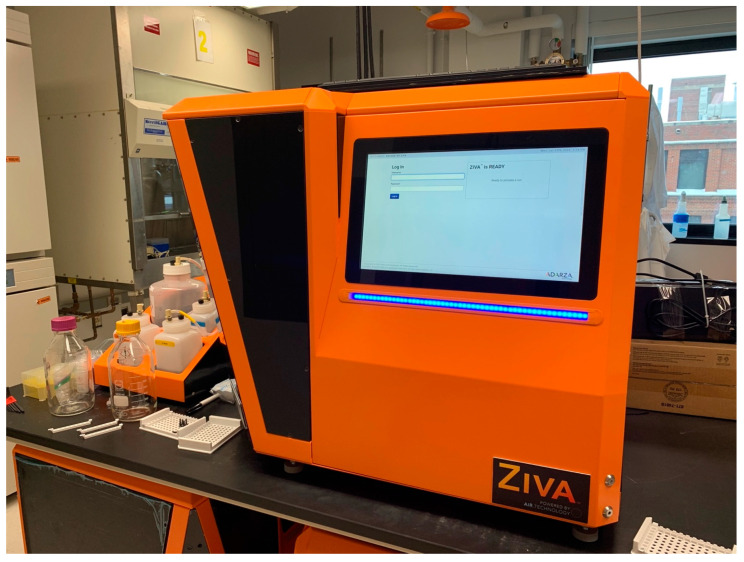
The ZIVA automated AIR platform produced by Adarza Biosystems, Inc., St. Louis, MO, USA.

Around the same time, commercialization of AIR was completed by Adarza BioSystems, Inc. Although it is no longer in operation, Adarza developed a high-throughput, fully automated version of AIR called “ZIVA” (Figure 2). This instrument brought all of the sensor washing, drying, and imaging steps of the AIR process into a single automated, internet-connected instrument. Building on the Miller group’s work described above, the first product available on ZIVA was an 18-plex respiratory pathogen antigen array. Initial qualification of ZIVA was accomplished using the same convalescent COVID-19 patient samples previously studied with “manual” AIR. Success with this array led to its expansion to 34-plex; this array included proteins from SARS-CoV-2, several other circulating coronaviruses, several strains of influenza, and respiratory syncytial virus (RSV).

The high-throughput capabilities of ZIVA make it a useful tool not only for testing individual antibody–antigen interactions but also as a research tool for understanding the relationships among antibody responses to all the antigens on the array [27]. In a study published in 2023, we used the automated ZIVA instrument with prefabricated 16- and 34-plex AIR arrays to study both convalescent and longitudinal samples [27]. Longitudinal samples followed individuals pre-SARS-CoV-2 vaccination through revaccination time points and for several months afterwards. Early experiments used a 16-plex respiratory antigen array. Samples run on this array were early nonconfirmed samples from individuals with respiratory illnesses in late 2019 and early 2020 as well as samples from convalescent COVID-19 patients. The results showed that convalescent samples showed antibody binding to SARS-2 antigens at higher amounts than to the nonconfirmed samples. All samples showed consistent but varied binding to influenza antigens, as different people may have been exposed to different strains at different times through influenza infection or vaccination. 

The second part of the paper studied 30 subjects who had blood drawn pre-COVID-19 and at time points throughout vaccination and postvaccination. The subject serum samples were run on a 35-plex ZIVA AIR array. Increases in antibody binding to SARS-CoV-2 antigens in response to COVID-19 vaccination was readily observed for all subjects, as was the decrease in antibody binding over time. Additionally, the high-throughput results of the array were used to study cross-reactivity in COVID-19 vaccination response and the changes in antibody binding to antigen mutations. Linear regressions were plotted for every combination of antigens on the array (561 combinations) to understand the relationships between antigen responses to vaccination. This showed that there is a strong linear relationship, indicated by the r-squared value, in the increase in antibody binding between SARS-CoV-2 receptor binding domain (RBD) and SARS-CoV-1 RBD (r^2^ = 0.83), as would be expected given the close similarity of these viruses. The relationship for SARS-CoV-2 S2 protein and the common cold hCoV 229E spike protein is less strong (r^2^ = 0.77) but still statistically significant, while the SARS-CoV-2 S1 and SARS-CoV-2 S1 D614G mutant have a stronger relationship (r^2^ = 0.93). The study also compared the waning of antibody binding to SARS-CoV-2 RBD protein vs. influenza California 07/2009 hemagglutinin protein, a pandemic influenza strain from 2009 which has also been included in several iterations of influenza vaccines. Samples from subjects who received the booster or had a breakthrough infection were also run on the 34-plex arrays to look at antibody binding. Lastly, the study also compared results of using diluted serum for sample incubation vs. whole blood from a finger prick from the same individual. It was found that 3 µL of whole blood produced comparable results to serum for antibody binding to antigens. 

## 6. StaphAIR

StaphAIR, an AIR array of *Staphylococcus aureus* (SA) antigens for detecting SA antibodies, was published in 2021 [12]. In the study, serum samples from 80 human subjects with musculoskeletal infections and 30 controls were used. A dilution series of pooled serum samples were incubated on staphAIR chips at 1 h at room temperature and overnight at 4 °C. The results of the experiment were plotted as thickness over concentration and fit to a 4pl model. These fit parameters were used to determine limit of detection (LOD) and limit of blank (LOB) for each antigen on the array. The LOB and LOD at 1 h incubation time was 1:100,000–1:10,000 dilution, while for the overnight time, the LOB and LOD decreased to 1:1,000,000. The experiment showed that an overnight incubation can extend the LOD by an order of magnitude compared to a 1 h room temperature incubation. Four SA antigens (IsdA, IsdB, Gmd, and SCIN) were found to be significantly different individually between SA-positive and SA-negative samples. IsdB was also found to be the antigen that best identified SA-positive infections correctly as found by a receiver operating characteristic (ROC) curve analysis. An “area under the curve of the receiver operating characteristic” (AUC ROC) analysis was performed on each antigen as well as on combinations of antigens. The outcome of the analysis found which combinations of antigens best helped distinguish SA-positive from SA-negative patients [12]. The strongest AUC, 0.861, came from a combination of biomarkers, IsdB, Amd, IsdA, SCIN, Hla. In comparison, the strongest AUC for a single antigen, IsdB, was 0.778. The results suggest that the use of StaphAIR in combination with multivariate analysis has promise as a potential tool for diagnosing musculoskeletal infections.

## 7. Aqueous AIR to Measure Kinetic Values of Splicing Factor Muscleblind-like Splicing Regulator 1 (MBNL1) with RNA Targets

**Figure 3:**
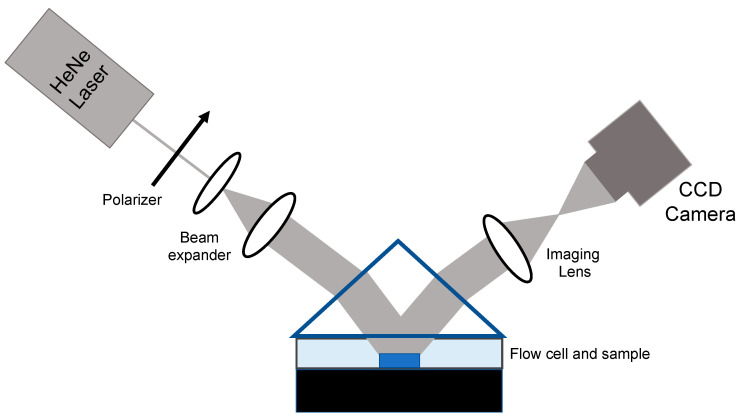
Schematic of the apparatus for aqueous AIR. A prism is used to couple light into and out of the flow cell.

Most experiments conducted with AIR have relied on rinsing and drying the sensor chip after a period of time in contact with the sample of interest; that time is chosen to optimize sensitivity while remaining cognizant of the need for efficient use of experimental time. AIR is not limited to a “dry” format, however, and AIR technology with flow has been used to measure kinetic constants [19]. To enable aqueous AIR, several adjustments to the standard AIR setup and protocol were implemented. Direct implementation of the AIR substrate under an aqueous cover material would require a glancing angle of incidence, creating significant imaging challenges. Therefore, to achieve the antireflective condition at a reasonable angle of incidence, the refractive index of the substrate was increased by fabricating a silicon/silicon nitride/silicon dioxide stack (Figure 3). This yielded an optimal angle of incidence for this substrate of 52.35°. Videos of the images were taken at three frames per minute. 

**Figure 4:**
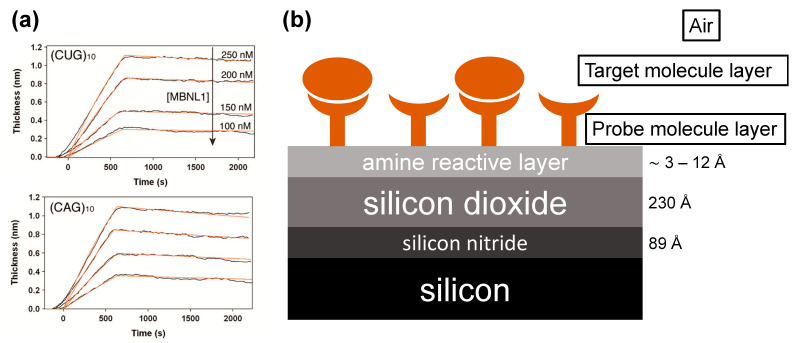
(**a**) Kinetic binding responses observed by aqueous AIR for MBNL1 interacting with two RNA hairpins. (**b**) Aqueous AIR requires an intermediate silicon nitride layer between the silicon substrate and silicon dioxide to attain the antireflective condition at a usable incident angle of light.

The method was tested in the context of measuring binding of various RNAs to MBNL1, an RNA splicing regulator. MBNL1 is known to bind with high affinity to repeat sequences, which can lead to dysregulation of splicing, which in turn can lead to type 1 myotonic dystrophy (DM1), among other disorders [28,29]. Several RNAs known to bind MBNL1 were tested in an array format, as was an HIV RNA known to not bind MBNL1 as a negative control. Kinetic on and off rates (k_a_ and k_d_, respectively) were determined by fitting data to a 1:1 Langmuir binding model. The kinetic values determined were consistent with values previously reported in the literature, measured using other, singleplex techniques. Representative traces obtained from single array spots are shown in Figure 4.

## 8. Using ZIVA for Equilibrium Antibody–Antigen Affinity Measurements

**Figure 5:**
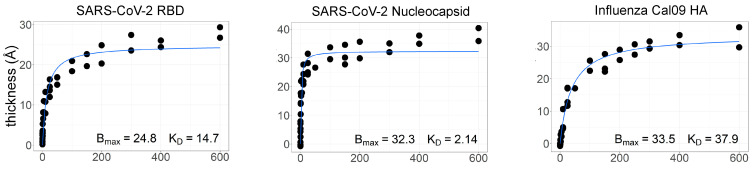
Plots of thickness (Å) vs. concentration of polyclonal antibody (µg/mL) for three respiratory virus proteins, SARS-CoV-2 RBD, SARS-CoV-2 nucleocapsid, and the influenza A California 2009 hemagglutinin (HA). Increase in thickness indicates an increase in binding. Bmax and K_D_ determined by fitting data to a one-site binding model. All concentrations in μg/mL.

While the work with aqueous AIR described above proved the utility of AIR for multiplex analysis of binding kinetics, we also set out to determine whether thermodynamic affinities could be measured using the “dry” version of the technique. The ZIVA instrument was used to find the affinity of protein–antibody interactions on the respiratory array using serial dilutions of commercially purchased polyclonal antibodies (Figure 5; see methods in Appendix A). Polyclonal antibodies tested were against the SARS-CoV-2 nucleocapsid protein, the SARS-CoV-2 RBD protein, and the hemagglutinin (HA) from the California 2009 pandemic H1N1 influenza strain. The thickness increase with increasing antibody concentration was plotted for each protein and fit to a one-site binding curve to model the binding interaction and obtain the equilibrium dissociation constant, K_D_. 

**Figure 6:**
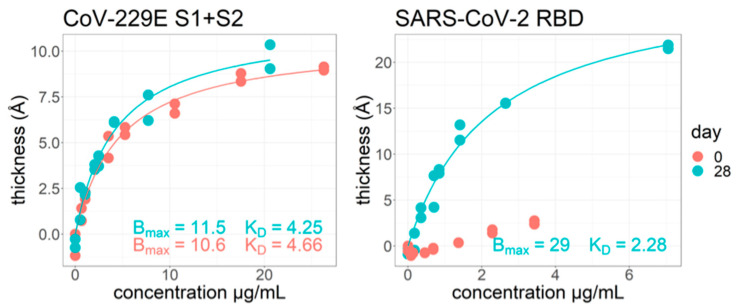
Plots of thickness (Å) vs. concentration of IgG purified serum. Data are for one subject (#144) of a study testing changes in antibody affinity (using AIR) for subjects after COVID-19 disease. No change was observed for CoV-229E S1+S2 protein (**left**), while a significant increase in affinity was observed for antibodies against SARS-CoV-2 RBD protein (**right**). Bmax and K_D_ determined by fitting data to a one-site binding model. The antibody response to SARS-CoV-2 RBD was too weak at day = 0 to determine a K_D_ value.

As part of a 2020 study on acutely infected symptomatic hospitalized COVID-19 patients [30], we also used ZIVA to investigate the difference in equilibrium affinity values on self-reported “Day 0” of infection and “Day 28.” (Figure 6). The concentration of antibodies in the samples was determined by ELISA. Samples were run at a range of concentrations on the ZIVA instrument. An example outcome is shown for sample #144. For the common cold viral spike protein “CoV-229E S1+S2”, there are already strong antibodies present on day 0. The Bmax and K_D_ values do not have a large change from day 0 to day 28. Since this is a common cold protein, we expected there to already be specific antibodies present at day 0. We also expected that the K_D_ would not change at day 28, because the infection is of SARS-CoV-2 and not the common cold. For the SARS-CoV-2 RBD protein, the day 0 response is mostly nonspecific binding increasing linearly, indicating the body has not produced specific antibodies yet against this protein. The day 28 response is different from day 0 in that it produces a specific response with a stronger K_D_ than for the common cold protein. This method has potential for simultaneously measuring equilibrium K_D_ against an array of proteins for a given sample. 

## 9. Conclusions

In the nearly two decades since its discovery, AIR has been used to detect a wide range of biomarkers in a broad set of human and animal samples. AIR has been shown with multiplexing up to hundreds of probes, but the technology has the capacity to be expanded to thousands, depending on substrate size, spot size, and the number of spot replicates. Results from the studies detailed above show utility in both clinical and research settings. AIR’s ability to sensitively detect molecules ranging from small molecules and bacteria, to cytokines, antibodies, and viruses shows its versatility, as does its ability to use capture molecules ranging from carbohydrates to nucleic acids to proteins. As with other label-free assays, detection of small molecules is more challenging than proteins or other large molecules. Competitive assays can enhance the sensitivity of small-molecule detection, as was shown in 2020 for the HIV protease inhibitor darunavir [16]. AIR’s strength continues to lie in the dynamic range of the technique, which ranges from small molecules to proteins hundreds of kiloDaltons in size. AIR has been particularly successful in antibody detection for various vaccine studies. AIR has been used as a biophysical tool to calculate affinity values at equilibrium and over time using aqueous AIR. The creation of a commercial product showed the full capabilities of AIR as a high-throughput tool to measure binding to many molecules at once and compare all these interactions quickly using computational tools. For all these reasons, AIR is poised to answer more questions useful in clinical and research settings as we move into the third decade of using this tool.

## Appendix A. Methods

### Appendix A.1. Antibodies/Convalescent Samples

Monoclonal antibodies were purchased from Sino Biological. Convalescent patient samples were purified for IgG antibody isotype. All participants were recruited at the University of Rochester Medical Center in Rochester, New York, and provided written informed consent prior to inclusion in these studies. These studies were approved by the University of Rochester Human Research Subject Review Board (protocol 14-0101) and conducted in accordance with Good Clinical Practice.

### Appendix A.2. ZIVA Experiments

ZIVA is a prototype instrument that automates washing and imaging steps for AIR arrays made by Adarza Biosystems, formerly in St. Louis, MO, USA. The ZIVA platform utilized 96-well plates of individually packaged, prearrayed AIR chips in custom-designed cartridges for sample addition. The arrays used in this study were the Acute Respiratory Virus Array (ARVA) kit arrays, also made by Adarza Biosystems. Each cartridge accepts 45 μL of solution. In this study, all serum samples were diluted 1:20 in assay wash buffer (AWB: mPBS with 0.005% tween-20, pH 7.2) containing 20% fetal bovine serum (FBS), which means that 3 μL of serum is required for the assay. The samples were added to the cartridges and allowed to incubate for 72 h at 4 degrees with shaking at 420 RPM. The plate was then loaded into the instrument and processed.

### Appendix A.3. ZIVA Analysis

The data were downloaded from the ZIVA platform as a .csv file. Data were processed using R [31]/Rstudio [32]. The Tidyverse [33] package was used to query, filter, and preprocess data. Plots with statistical tests were made with ggplot. Thicknesses on each chip were subtracted from a negative control chip (baseline thickness). Increase in binding over increase in concentration of sample was modeled with a one-site binding curve. Curves in ggplot were created using the ggpmisc package [34].
